# Eigenvibrations of Kirchhoff Rectangular Random Plates on Time-Fractional Viscoelastic Supports via the Stochastic Finite Element Method

**DOI:** 10.3390/ma16247527

**Published:** 2023-12-06

**Authors:** Marcin Kamiński, Michał Guminiak, Agnieszka Lenartowicz, Magdalena Łasecka-Plura, Maciej Przychodzki, Wojciech Sumelka

**Affiliations:** 1 Department of Structural Mechanics, Faculty of Civil Engineering, Architecture & Environmental Engineering, Łódź University of Technology, Al. Politechniki 6, 90-924 Łódź, Poland; 2 Institute of Structural Analysis of Poznan University of Technology, Piotrowo 5 Street, 60-965 Poznan, Poland; michal.guminiak@put.poznan.pl (M.G.); magdalena.lasecka-plura@put.poznan.pl (M.Ł.-P.); maciej.przychodzki@put.poznan.pl (M.P.); wojciech.sumelka@put.poznan.pl (W.S.); 3 Doctoral School of Poznan University of Technology, Piotrowo 3 Street, 60-965 Poznan, Poland; agnieszka.z.lenartowicz@doctorate.put.poznan.pl

**Keywords:** stochastic finite element method, free damped vibrations, Kirchhoff–Love plates, viscoelastic dampers, fractional derivatives, continuation method, semi-analytical probabilistic technique, stochastic perturbation technique, Monte-Carlo simulations, probabilistic relative entropy, reliability analysis

## Abstract

The present work’s main objective is to investigate the natural vibrations of the thin (Kirchhoff–Love) plate resting on time-fractional viscoelastic supports in terms of the Stochastic Finite Element Method (SFEM). The behavior of the supports is described by the fractional order derivatives of the Riemann–Liouville type. The subspace iteration method, in conjunction with the continuation method, is used as a tool to solve the non-linear eigenproblem. A deterministic core for solving structural eigenvibrations is the Finite Element Method. The probabilistic analysis includes the Monte-Carlo simulation and the semi-analytical approach, as well as the iterative generalized stochastic perturbation method. Probabilistic structural response in the form of up to the second-order characteristics is investigated numerically in addition to the input uncertainty level. Finally, the probabilistic relative entropy and the safety measure are estimated. The presented investigations can be applied to the dynamics of foundation plates resting on viscoelastic soil.

## 1. Introduction

Some stochastic numerical approaches, especially the Boundary and Finite Element Methods, have been intensively studied by many authors. Kamiński et al. applied the Finite Element Method (FEM) for the stochastic second-order perturbation technique [1] as well as the iterative scheme in the determination of the probabilistic moments of the structural response [2]. Cheng et al. performed reliability analysis of plane elasticity problems via stochastic spline fictitious BEM [3] and a similar investigation for Reissner’s plate bending problem [4] and fracture mechanics analysis of linear-elastic cracked structures [5]. Christos et al. [6] used the Stochastic Boundary Element Method (SBEM) to analyze the dynamic response of tunnels. Karimi et al. [7] proposed coupled FEM-BEM with artificial neural networks (ANN) approach for the system identification of concrete gravity dams. A coupled FEM-BEM-least squares point interpolation for a structure-acoustic system with a stochastic perturbation technique was proposed by Zhang et al. [8]. The stochastic Galerkin scaled boundary finite element approach was proposed by Duy Mihh et al. [9] for the randomly defined domain. The Monte Carlo simulation technique coupled with the scaled boundary FEM was applied by Chowdhury et al. [10] for probabilistic fracture mechanics. Similarly, the same authors studied probabilistic fracture mechanics with uncertainty in the crack size and orientation in terms of the scaled boundary finite element approach [11]. Luo et al. investigated the stochastic response determination of multidimensional nonlinear systems endowed with fractional derivatives [12].

Di Matteo et al. studied stochastic response determination of nonlinear oscillators with fractional derivatives elements via the Wiener path integral [13]. Łasecka-Plura and Lewandowski [14] investigated dynamic characteristics and frequency response function for frames with dampers using FEM with uncertain design parameters. Abdelrahman et al. studied the nonlinear dynamics of viscoelastic flexible structural systems using the finite element method [15]. Ratas et al. investigated the solution of the nonlinear boundary value problems by applying the higher-order Haar wavelet functions [16], where the authors carried out a comprehensive analysis of the error and convergence of the obtained solution. Abdelfattah et al. investigated the generalized nonlinear quadrature for the fractional-order chaotic systems using the SINC shape function [17] in which the authors applied the generalized Caputo definition of the fractional derivative to a study of fractional-order Lorenz oscillator describing a three-dimensional chaotic flow dynamical system.

The basis for considerations in the random approach is a finite set of solutions in the deterministic approach. The problem of deterministic damped vibrations employing viscoelastic dampers was the subject of Lewandowski’s [18] and Pawlak with Lewandowski [19]’s research. Similarly, the dynamic characteristics of multilayered beams with viscoelastic layers described using the time-fractional Zener model have been investigated by Lewandowski and Baum [20]. Łasecka-Plura and Lewandowski [21] applied the subspace iteration method to resolve nonlinear eigenvalue problems occurring in the dynamics of structures with viscoelastic elements.

Material parameters of the viscoelastic constraints may be easily described using fractional derivatives [22]. Chang and Singh [23] investigated the seismic analysis of structures with a fractional derivative model of viscoelastic dampers. On the other hand, Kun et al. [24] applied fractional derivatives to analyze the stochastic seismic response of structures considering viscoelastic dampers. Next, Shubin [25] applied the generalized multiscale finite element method to an inverse random source problem. It is important to emphasize that all papers mentioned above studied the time-fractional viscoelastic dampers utilizing the “full memory” approach [22,25,26], i.e., the fractional operator governs the memory from initial time up to current time. Such an assumption may be generalized by using the “short memory” approach, in which memory is restricted to a particular time interval (time length scale) [22,27,28].

The present work’s main objective is to investigate the natural vibrations of the thin (Kirchhoff–Love) plate resting on the time-fractional viscoelastic supports in terms of the Stochastic Finite Element Method (SFEM). The least squares method procedure enables the determination of random polynomials of eigenfrequencies, whose further integration with the Gaussian kernel returns probabilistic characteristics. Additionally, the stochastic perturbation technique (SPT) and the Monte Carlo simulation (MCS) approaches are all applied in the analysis to study the influence of various uncertainty sources on the basic eigenfrequencies of some more popular rectangular plates with viscoelastic supports.

## 2. Eigenvibrations Analysis Methodology of Deterministic Approach

The presented investigations aim to determine the first few natural frequencies of thin rectangular elastic and isotropic plates supported on the boundary and rested on the (classical) viscoelastic dampers.

A thin, elastic, and isotropic plate is considered, which can be supported classically in an ideal way, e.g., along the shore, and can also rest on a finite number of supports that have the nature of viscoelastic constraints. The plate may also rest solely on viscoelastic supports, which may be located on its edges or inside it. Due to the discrete and one-dimensional nature of additional supporting viscoelastic bonds, it is very easy to take into account their presence in the description of the deformation of the entire structural system, i.e., the plate-viscoelastic constraints. Thus, the presence of viscoelastic elements is taken into account in the boundary conditions while ensuring the kinematic invariance of the system. Applying the classical variational formulation to a plate single finite element, one obtains, e.g., [29].

(1) $$\int_{V}\delta u^{T}FdV+\int_{S}\delta u^{T}\Phi dS+\sum_{i=1}^{n}\delta {\left(u^{T}\right)}_{i}p_{i}=\int_{V}\delta \varepsilon^{T}\sigma dV+\int_{V}\delta u^{T}\rho \ddot{u}dV+\int_{V}\delta u^{T}k_{d}\dot{u}dV$$

where *δ***u** and *δ***ε** are column matrices of variations of small displacements and strains; **F** is the vector of volumetric forces (body forces); **Φ** is the column matrix of tractions acting on the surface; **p***_i_* is the vector of concentrated loads acting at a selected point “*i*”; *k_d_* is a viscosity damping parameter; and finally, $\dot{u}$ and $\ddot{u}$ are the velocity and acceleration vectors, respectively [29]. Equation (1) can be generalized and written for a set of finite elements, whereby the actual damping elements (viscoelastic elements) will be coupled with translational displacements perpendicular to the center surface of the plate. The above general formulation allows for a simple way of writing down the problem of system vibrations in a matrix manner by the FEM methodology. A description of the finite element analysis approach is provided below.

**Figure 1:**
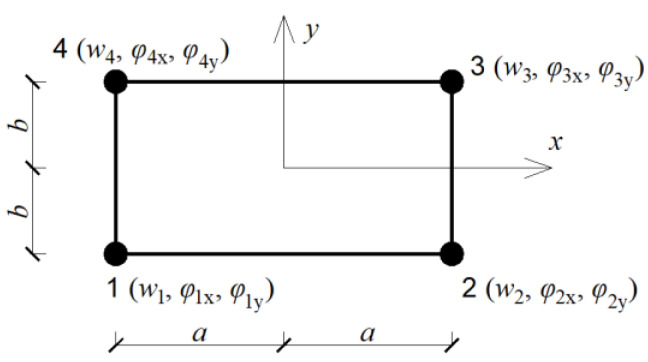
Finite element used for discretization of tested plate: node numbering and active degrees of freedom.

The Finite Element Method (FEM) with a regular discretization including 4-noded plate finite elements is applied to the numerical analysis (Figure 1).

The displacement vector $w_{i}^{e}$ of the i-th corner node within the finite element e is defined as [30].

(2) $$w_{i}^{e}={\left[\begin{array}{ccc} w_{i} & \varphi_{ix} & \varphi_{iy} \end{array}\right]}^{T}={\left[\begin{array}{ccc} w_{i} & \frac{\partial w_{i}}{\partial y} & -\frac{{\partial w}_{i}}{\partial x} \end{array}\right]}^{T};i=1,2,3,4.$$

**Figure 2:**
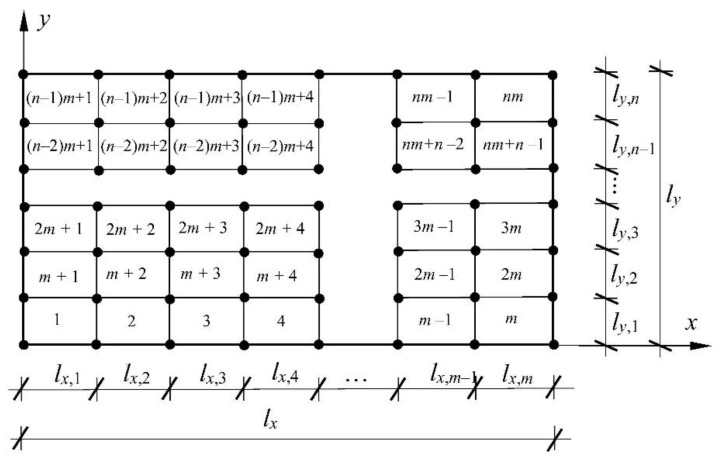
Plate discretization with rectangular plate finite elements.

The field of displacements inside the element e is expressed as a linear combination of the shape functions $N_{k}^{e}(x,y)$.

(3) $$w^{e}\left(x,y\right)=N_{e}w_{e},$$

where $w_{e}={\left[w_{1}^{e},w_{2}^{e},w_{3}^{e},w_{4}^{e}\right]}^{T}$ and $N_{e}=\left[N_{1}^{e},N_{2}^{e},N_{3}^{e},\ldots N_{12}^{e}\right]$. The shape functions, the element stiffness matrix $K_{e}$ and the element consistent mass matrix $M_{e}$ are defined according to the standard FEM methodology [29,31]. To conclude, the stiffness matrix is derived analytically, whereas the mass matrix is calculated numerically using 16th point Gaussian quadrature. The discretization of a plate domain is shown in Figure 2, where the adopted method of numbering finite elements and their nodes is presented. The center plane of the plate with dimensions A×B is discretized with m×n finite elements with dimensions $l_{x,i}\times l_{y,j}$ (i=1,2,3,…,m;j=1,2,3,…,n). It was assumed that all finite elements have the same dimensions $l_{x}\times l_{y}$. The discretized plate contains a total of $\left(m+1\right)(n+1)$ nodes and $3\left(m+1\right)(n+1)$ degrees of freedom.

The matrix equation of the force balance for a structure with viscoelastic dampers can be written in the following form:

(4) $$M\ddot{q}\left(t\right)+C\dot{q}\left(t\right)+Kq\left(t\right)=f\left(t\right)+p\left(t\right),$$

where **M** and **K** denote mass and stiffness matrices of the plate, respectively; matrix C denotes the global plate damping matrix; $f\left(t\right)$ is the vector of additional forces resulting from the viscoelastic supports; and $p\left(t\right)$ is the excitation vector.

An application of the Laplace transform with zero initial conditions leads to the following transform of Equation (4)

(5) $$\left(s^{2}M+sC+K\right)\overset{-}{q}\left(s\right)=\overset{-}{f}\left(s\right)+\overset{-}{p}\left(s\right),$$

where $\overset{-}{q}\left(s\right)$ is the L-transform of $q\left(t\right)$, $\overset{-}{p}\left(s\right)$ is the L-transform of $p\left(t\right)$ and the vector $\overset{-}{f}\left(s\right)$ is expressed as

(6) $$\overset{-}{f}\left(s\right)=-\sum_{r=1}^{n_{d}}\left(K_{r}+G_{r}(s)\right)L_{r}\overset{-}{q}\left(s\right).$$

In Equation (6), $n_{d}$ denotes the total number of dampers attached to the plate at selected nodes of a finite element mesh and $L_{r}$ is a global matrix indicating the location of the r-th damper within the plate domain.

**Figure 3:**
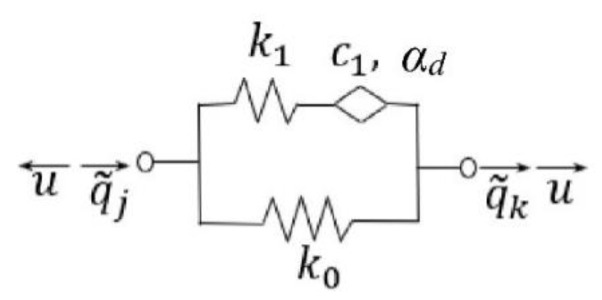
The model of viscoelastic damper.

A viscoelastic damper is described graphically in Figure 3, wherein *k*_0_, *k*_1_, *c*_1_, and *α_d_* describe material properties of viscoelastic constraints (damper); *u* is the force transferred by the damper; and ${\tilde{q}}_{j}$ and ${\tilde{q}}_{k}$ are displacements occurring at the end of viscoelastic constraints.

Consequently, quantities appearing in Equation (6) can be expressed as follows:

(7) $$K_{r}=k_{0};\;G_{r}\left(s\right)=\frac{k_{1j}s^{\left(\alpha_{d}\right)}}{\nu_{1j}+s^{\left(\alpha_{d}\right)}},$$

where $\nu_{1j}=k_{1j}/c_{1j}$ is the quotient of the stiffness and damping coefficients of the j-th damper element.

Next, let the excitation vector $p\left(t\right)$ to be equal to zero. Hence, the relation described by Equation (4) takes the following form:

(8) $$M\ddot{q}\left(t\right)+C\dot{q}\left(t\right)+Kq\left(t\right)=f\left(t\right).$$

Now, the Laplace transform of Equation (8) leads to the following relation:

(9) $$\left(s^{2}M+sC+K+K_{d}+G_{d}(s)\right)\overset{-}{q}\left(s\right)=0,$$

where

(10) $$K_{d}=\sum_{r=1}^{n_{d}}K_{r}L_{r},$$

(11) $$G_{d}\left(s\right)=\sum_{r=1}^{n_{d}}G_{r}\left(s\right)L_{r}.$$

Equation (9) describes a nonlinear eigenproblem that is solved for the eigenvalues s and the eigenvectors $\overset{-}{q}\left(s\right)$ using the subspace iteration method coupled with the continuation method. The continuation method was comprehensively described by Lewandowski in [20].

The obtained eigenvalues of Equation (9) are complex numbers of the form $s_{j}=\mu_{j}+i\eta_{j}$. This is the basis for the j-th natural frequency $\omega_{j}$ of the structure and the non-dimensional damping ratio $\gamma_{j}$ of the j-th mode of vibration:

(12) $$\omega_{j}^{2}=\mu_{j}^{2}+\eta_{j}^{2},\;\gamma_{j}=-\frac{\mu_{j}}{\omega_{j}}.$$

The subspace iteration method makes it possible to calculate the first few natural frequencies and the corresponding non-dimensional damping ratios without solving the entire nonlinear eigenproblem. A general description of the subspace iteration method is given below—cf. [21].

A nonlinear eigenproblem (Equation (9)) can be rewritten as

(13) $$MX\Lambda^{2}+CX\Lambda +KX+K_{d}X+\sum_{r=1}^{n_{d}}L_{r}X\Gamma_{r}\left(s\right)=0$$

where $X=\left[{\overset{-}{q}}_{1},{\overset{-}{q}}_{2},\ldots ,{\overset{-}{q}}_{n}\right]$, $\Lambda =diag\left(s_{1},s_{2},\ldots ,s_{n}\right)$, $\Gamma_{r}\left(s\right)=diag\left(G_{r}\left(s_{1}\right),G_{r}\left(s_{2}\right),\ldots ,G_{r}\left(s_{n}\right)\right)$ and *n* denotes the number of degrees of freedom of the considered structure.

The first step is to find a solution of a linear eigenproblem in the following form:

(14) $$\left(\left(K+K_{d}\right)-\omega^{2}M\right)x=0.$$

The solution Equation (14) consists of *n* real eigenvalues and corresponding eigenvectors. Then, the first $\tilde{m}$ modes are taken as the initial approximation:

(15) $$\Lambda_{0}=diag\left(s_{1}^{\left(0\right)},s_{2}^{\left(0\right)},\ldots ,s_{\tilde{m}}^{\left(0\right)}\right),\;X_{0}=\left[{\overset{-}{q}}_{1}^{\left(0\right)},{\overset{-}{q}}_{2}^{\left(0\right)},\ldots ,{\overset{-}{q}}_{\tilde{m}}^{\left(0\right)}\right]$$

where $s_{j}^{\left(0\right)}=0+i\omega_{j}$ and ${\overset{-}{q}}_{j}^{\left(0\right)}=x_{j}+i0$ for $j=1,2,\ldots ,\tilde{m}$. In subsequent loops of the subspace iteration method, first, the following equation is solved with respect to ${\tilde{X}}_{k}$:

(16) $$\left(K+K_{d}\right){\tilde{X}}_{k}=P_{k-1}$$

where $P_{k-1}=-MX_{k-1}\Lambda_{k-1}^{2}-CX_{k-1}\Lambda_{k-1}-\sum_{r=1}^{nd}L_{r}X_{k-1}\Gamma_{k-1,r}\left(s^{\left(k-1\right)}\right)$ and *k* is the number of iterations. Next, the reduced nonlinear eigenproblem has to be solved:

(17) $$\left({s^{\left(k\right)}}^{2}{\tilde{M}}_{k}+s^{\left(k\right)}{\tilde{C}}_{k}+\left({\tilde{K}}_{k}+{\tilde{K}}_{dk}\right)+{\tilde{G}}_{k}\left(s^{\left(k\right)}\right)\right)z^{\left(k\right)}=0$$

where ${\tilde{M}}_{k}={\tilde{X}}_{k}^{H}M{\tilde{X}}_{k}$, ${\tilde{C}}_{k}={\tilde{X}}_{k}^{H}C{\tilde{X}}_{k}$, ${\tilde{K}}_{k}={\tilde{X}}_{k}^{H}K{\tilde{X}}_{k}$, ${\tilde{K}}_{dk}={\tilde{X}}_{k}^{H}K_{d}{\tilde{X}}_{k}$, ${\tilde{G}}_{k}\left(s^{\left(k\right)}\right)=\sum_{r=1}^{nd}{\tilde{X}}_{k}^{H}L_{r}{\tilde{X}}_{k}G_{r,k}\left(s^{\left(k\right)}\right)$. The solution of Equation (17) is a new approximation of $\tilde{m}$ eigenvalues $s_{j}^{\left(k\right)}$ and eigenvectors $z_{j}^{\left(k\right)}$. The nonlinear Hermitian eigenproblem (Equation (17)) is solved by the continuation method [21], which is successfully used in the analysis of systems with viscoelastic elements [19,20].

A new approximation of eigenvectors of the origin nonlinear eigenproblem (Equation (9)) is computed as follows:

(18) $$X_{k}={\tilde{X}}_{k}Z_{k}$$

where $Z_{k}$ is formed as $Z_{k}=\left[z_{1}^{\left(k\right)},z_{2}^{\left(k\right)},\ldots ,z_{\tilde{m}}^{\left(k\right)}\right]$. The iterative process is completed when the following conditions are fulfilled:

(19) $$\left|s_{j}^{\left(k\right)}-s_{j}^{\left(k-1\right)}\right|\leq \varepsilon_{1}\left|s_{j}^{\left(k\right)}\right|,\;\;\;\left\|{\overset{-}{q}}_{j}^{\left(k\right)}-{\overset{-}{q}}_{j}^{\left(k-1\right)}\right\|\leq \varepsilon_{2}\left\|{\overset{-}{q}}_{j}^{\left(k\right)}\right\|\;for\;j=1,2,\ldots ,\tilde{m}$$

where $\varepsilon_{1}$ and $\varepsilon_{2}$ are the assumed accuracies of calculations. In the current work, the required accuracy for eigenvalues and eigenvectors is 10^−4^.

As mentioned above, the time-fractional viscoelastic support description is considered. The one-dimensional constitutive equation for viscoelastic constraints is introduced to the analysis [32]

(20) $$\sigma \left(t\right)+\tau^{\alpha_{d}}\frac{d^{\alpha_{d}}\sigma \left(t\right)}{dt^{\alpha_{d}}}=E_{0}\varepsilon \left(t\right)+\tau^{\alpha_{d}}E_{0\infty }\frac{d^{\alpha_{d}}\varepsilon \left(t\right)}{dt^{\alpha_{d}}},$$

where σ and ε are the stress and the strain, *E*_0_ and *E*_∞_ are the relaxed and non-relaxed elastic moduli, and τ is the relaxation time.

Compared with the classical Zener model of damper, in the model shown in Figure 3, the element with only damping properties is replaced by a viscoelastic element, also the so-called Scott–Blair element (in Figure 3 shown as a rhombus). It is described by two parameters: *c*_1_—damping coefficient and *α^d^*—order of the fractional derivative $\left(0<\alpha^{d}\leq 1\right)$.

The constitutive equation for the Scott-Blair element can be written as

(21) $$u\left(t\right)=c_{1}D_{t}^{\alpha_{d}}\Delta q\left(t\right),$$

where $D_{t}^{\alpha_{d}}\left(•\right)$ denotes the fractional derivate of the order $\alpha_{d}$ with respect to the time and $\Delta q\left(t\right)={\tilde{q}}_{k}-{\tilde{q}}_{j}$. In this paper, the Riemann–Liouville definition [22,32] is applied, which is mainly used for the description of viscoelastic dampers [18,23,24]:

(22) $$D_{t}^{\alpha_{d}}f\left(t\right)=\frac{df\left(t\right)}{dt^{\alpha^{d}}}=\frac{1}{Г\left(1-\alpha^{d}\right)}\frac{d}{dt}\int_{0}^{t}\frac{f\left(s\right)}{{\left(t-s\right)}^{\alpha_{d}}}ds$$

## 3. Structural Response Recovery and the Probabilistic Analysis

The eigenfrequencies $\omega_{i}$ of the plate under consideration have been all found via the polynomial basis

(23) $$\omega_{i}=\sum_{j=0}^{n}C_{ij}\nu^{j}$$

Applying the least squares method (LSM) based on several FEM deterministic solutions for varying values of the Gaussian input uncertainty source is denoted by *ν*. Then, approximations of various orders are subjected to some optimization procedure, where the deterministic search method enables a choice of the optimal polynomial, which minimizes the mean square error and maximizes the correlation coefficient for the structural output and input.

The basic probabilistic characteristics such as expectations, coefficients of variation, skewness, and kurtosis have been estimated via application of integral definitions:

(24) $$E\left[\omega_{i}\right]=\int_{-\infty }^{+\infty }\sum_{j=1}^{n}C_{ij}v^{j}p_{v}(x)dx,\alpha \left(\omega_{i}\right)=\left|\frac{\sigma \left(\omega_{i}\right)}{E\left[\omega_{i}\right]}\right|,\;\beta \left(\omega_{i}\right)=\frac{\mu_{3}\left(\omega_{i}\right)}{\sigma^{3}\left(\omega_{i}\right)},\kappa \left(\omega_{i}\right)=\frac{\mu_{4}\left(\omega_{i}\right)}{\sigma^{4}\left(\omega_{i}\right)}.$$

Moreover, let *R* denote the admissible limit of the given structure, and *E* denote its extreme effort. The previous engineering designing codes allow us to make the following interpretation in case of the eigenfrequency and extreme excitation: a distance in-between them cannot be smaller than a quarter of this eigenfrequency. This is to avoid a structural resonance. Therefore, the satisfactory safety of the given system may be measured with the use of the following FORM reliability index [33]:

(25) $$\beta_{\mathrm{FORM}}=\frac{E\left[R\right]-E\left[E\right]}{\sqrt{Var\left(R-E\right)}}=\frac{E\left[\omega_{i}\right]-E\left[\frac{3}{4}\omega_{i}\right]}{\sigma \left(\omega_{i}-\frac{3}{4}\omega_{i}\right)},$$

where $\omega_{i}$ stands for each next eigenfrequency. Quite a similar calculation can be provided with the use of a relative entropy *H* quantifying a distance in-between two random distributions, which can be calculated due to the Bhattacharyya [34,35] theory as

(26) $$H=\frac{1}{4}\frac{{\left(E\left[R\right]-E\left[E\right]\right)}^{2}}{\sigma^{2}\left(R\right)+\sigma^{2}\left(E\right)}+\frac{1}{2}\ln\left(\frac{\sigma^{2}\left(R\right)+\sigma^{2}\left(E\right)}{2\sigma \left(R\right)\sigma \left(E\right)}\right).$$

The above relation can be rewritten as

(27) $$H\left(\omega_{i}\right)=\frac{1}{4}\frac{{\left(E\left[\omega_{i}\right]-E\left[\frac{3}{4}\omega_{i}\right]\right)}^{2}}{\sigma^{2}\left(\omega_{i}\right)+\sigma^{2}\left(\frac{3}{4}\omega_{i}\right)}+\frac{1}{2}\ln\left(\frac{\sigma^{2}\left(\omega_{i}\right)+\sigma^{2}\left(\frac{3}{4}\omega_{i}\right)}{2\sigma \left(\omega_{i}\right)\sigma \left(\frac{3}{4}\omega_{i}\right)}\right).$$

Then, the final safety measure compensable to *β*_FORM_ is obtained here as

(28) $$\beta_{H}=\frac{1}{2}\sqrt{H\left(\omega_{i}\right)}.$$

## 4. Numerical Examples

Four square plates will be analyzed: the first one with asymmetric boundary conditions and asymmetrically placed viscoelastic supports; the second one, which is simply supported on three adjacent edges, with the fourth edge resting on viscoelastic constraints; the third one, which is simply supported on two opposite edges with the other two resting on viscoelastic constraints; and the fourth one, with all edges resting on viscoelastic constraints. Random moments and reliability measures for the appropriate random variables will be estimated for all plates.

### 4.1. Plate 1

**Figure 4:**
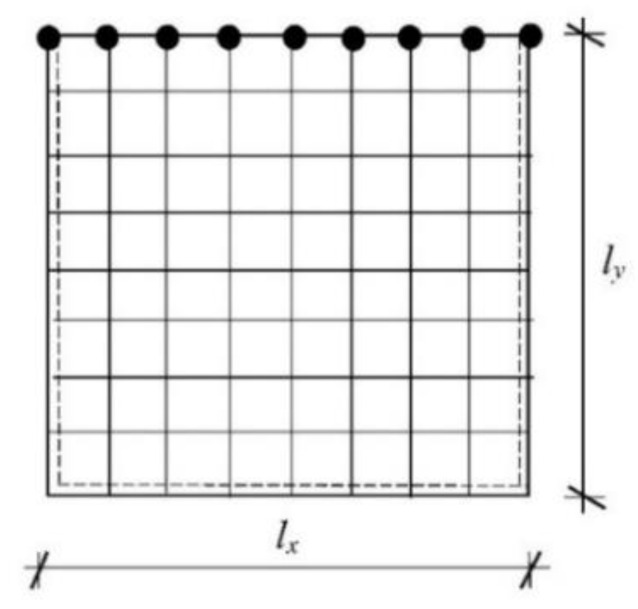
Finite element mesh of the square isotropic plate simply supported on three adjacent edges, with the fourth edge resting on viscoelastic constraints.

The square isotropic plate fixed along the right vertical edge and simply supported along the horizontal lower edge has been discretized using the 15 × 15 plate rectangular finite elements, whose material properties are *E* = 205 GPa, *ν*_p_ = 0.3, and *ρ*_p_ = 7850 kg/m^3^. The plate dimensions are equal to $l_{x}\times l_{y}\times H=\left(2.0\times 2.0\times 0.01\right)[m$]. The set of viscoelastic supports (dampers) have been attached along one plate edge (Figure 4). Structural damping is neglected. The first four modes have been analyzed. It was assumed that the required accuracy for eigenvalues and eigenvectors is 10^−4^.

Probabilistic computations are carried out for material properties of the plate and parameters describing viscoelastic supports whose values change randomly. The plate Young’s modulus ranges from 180 to 230 kN/m^2^ with an increase of 5 kN/m^2^, and the plate Poisson’s ratio varies from 0.25 to 0.35 with an increase of 0.01. The parameters describing the viscoelastic constraints change as follows: *k*_0_ ranges from 95 to 125 N/m with the increase of 3 N/m, *k*_1_ ranges from 17,500 to 22,500 N/m with the increase of 500 N/m, *c*_1_ ranges from 205 to 255 N·s/m with the increase of 5 N·s/m and finally *α_d_* ranges from 0.5 to 0.7 with the increase of 0.02. At the very beginning, the set of deterministic solutions for the first four natural circular frequencies and coefficients of damping is given; the results are summarized in Table 1 and Table 2, respectively.

The sample polynomial response functions for the first natural frequency and coefficient of damping have been obtained as the third-order polynomials listed below:

(29) $$\omega_{1}\left(E\right)=17.62484638948+0.196996865217006\cdot E+\;-0.000310522127550886\cdot E^{2}+2.9961147203988\cdot 10^{-7}\cdot E^{3},$$

(30) $$\gamma_{1}\left(E\right)=0.121979692682340-0.000663570090989016\cdot E+\;+1.92531471231778\cdot 10^{-6}\cdot E^{2}-2.2035742474593\cdot 10^{-9}\cdot E^{3},$$

**Figure 5:**
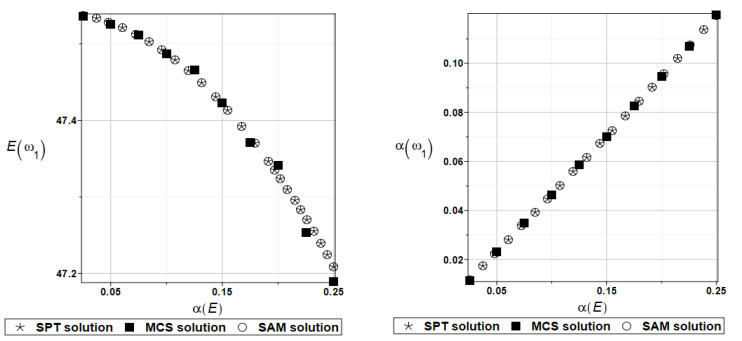
Probabilistic moments for the first natural frequency and the random distribution of Young’s modulus.

**Figure 6:**
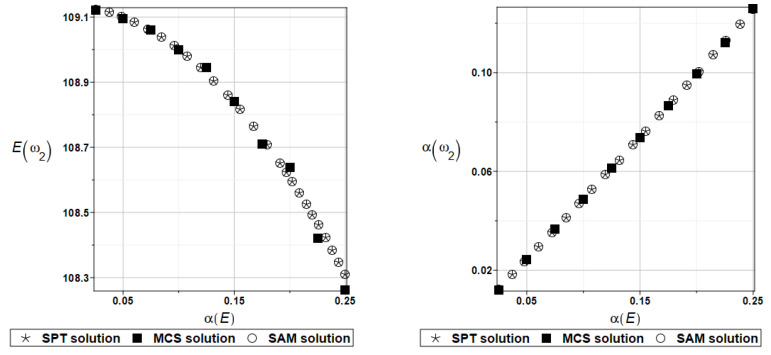
Probabilistic moments for the second natural frequency and the random distribution of Young’s modulus.

The expected value $E\left(\omega_{i}\right)$ and coefficient of variation $\alpha \left(\omega_{i}\right)$ of the first two natural frequencies (*i* = 1, 2) have been presented all in turn in Figure 5 and Figure 6, both as the functions of the input random modulus coefficient of variation. How it could be expected from the data collected in Table 1, an impact of the input uncertainty on the expectations of the first eigenfrequency, has a rather limited character. The output uncertainty, although linearly dependent upon the input one, is definitely damped by this system (almost two times).

**Figure 7:**
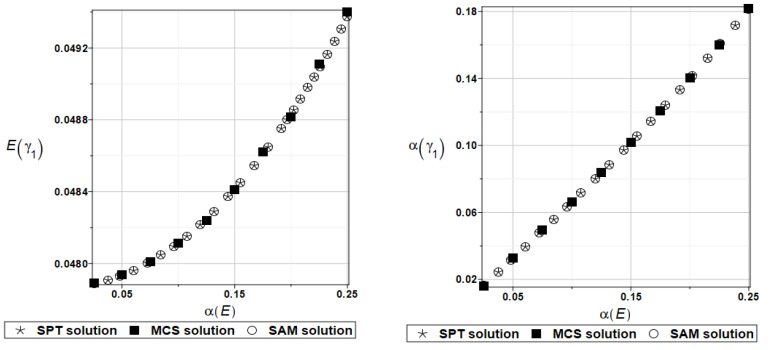
Probabilistic moments for the first coefficient of damping and the random distribution of Young’s modulus.

**Figure 8:**
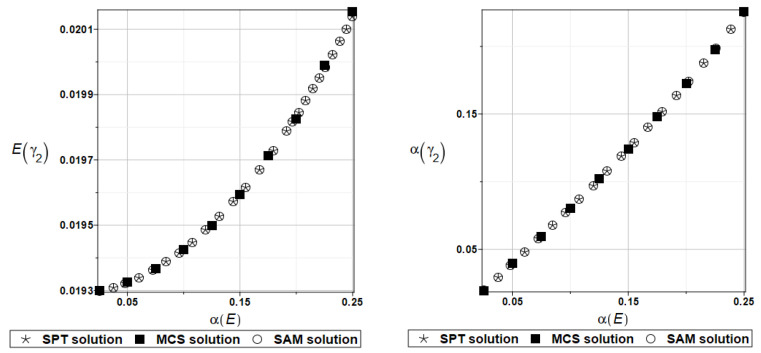
Probabilistic moments for the second coefficient of damping and the random distribution of Young’s modulus.

The expected value $E\left(\gamma_{i}\right)$ and coefficient of variation $\alpha \left(\gamma_{i}\right)$ of the first two natural frequencies (*I* = 1, 2) are presented in turn in Figure 7 and Figure 8. Contrary to the previous two figures, the expected values increase together with the input uncertainty level. All three numerical methods coincide perfectly for the entire range of fluctuations of the input coefficient of variation.

Similarly, as above, the set of deterministic solutions for the first four natural circular frequencies and coefficients of damping is given. The results are summarized in Table 3 and Table 4, respectively.

**Figure 9:**
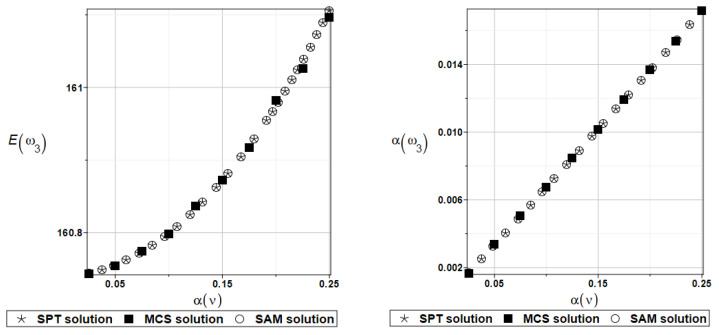
Probabilistic moments for the third natural frequency and the random distribution of Poisson’s ratio.

**Figure 10:**
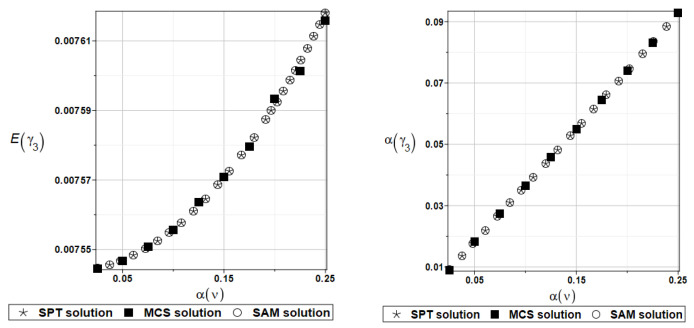
Probabilistic moments for the first coefficient of damping and the random distribution of Poisson’s ratio.

There is no doubt that Poisson’s ratio uncertainty induces almost no uncertainty in neither the first natural frequency nor in the coefficients of damping. This is confirmed in Figure 9 and Figure 10, where expected values $E\left(\omega_{i}\right)$ and coefficients $\alpha \left(\omega_{i}\right)$ of the circular frequencies (*i* = 3) and $E\left(\gamma_{i}\right)$, $\alpha \left(\gamma_{i}\right)$ are presented. Even with marginal resulting uncertainty, a coincidence of all three probabilistic numerical strategies is perfect for the first two moments.

Random distribution of the damper’s parameter *k*_0_ is considered further, and the set of deterministic solutions for the first four natural circular frequencies and coefficients of damping is given in Table 5 and Table 6 below. Is it clearly seen in any column that the impact of this parameter statistical scattering is negligible; the same holds true for the damper’s parameter *k*_1_; see Table 7 and Table 8.

**Figure 11:**
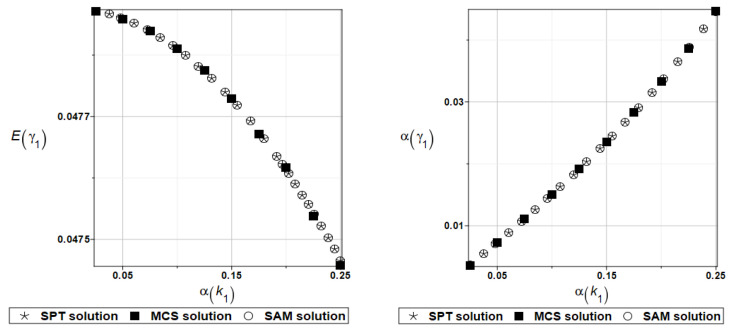
Probabilistic moments for the first coefficient of damping and the random distribution of the damper’s parameter k_1_.

A negligible sensitivity of the solution value to any change in the design parameter for the first four natural frequencies can be observed here, and therefore, the first two probabilistic moments: $E\left(\gamma_{i}\right)$ and $\alpha \left(\gamma_{i}\right)$ (*i* = 1) have been presented in turn in Figure 11.

Similarly, as in previous computations, the set of deterministic solutions for the first four natural circular frequencies and coefficients of damping is given. In this case, the sensitivity of the first two natural frequencies and all first four coefficients of damping to a change in the *c*_1_ parameter can be observed; the results are summarized in Table 9 and Table 10, respectively.

**Figure 12:**
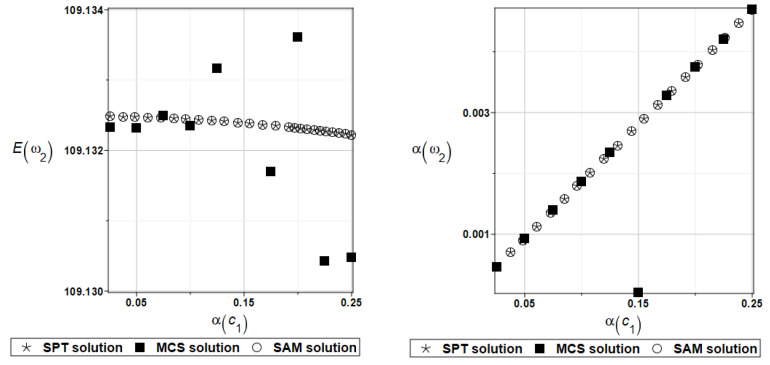
Probabilistic moments for the second natural frequency and the random distribution of the damper’s parameter *c*_1_.

**Figure 13:**
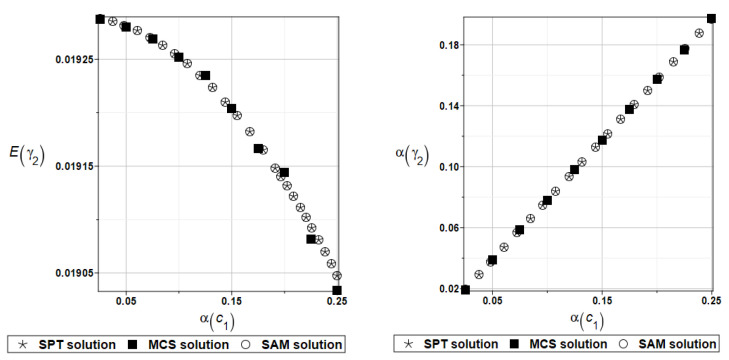
Probabilistic moments for the coefficient of damping and the random distribution of the damper’s parameter *c*_1_.

It is possible to observe the practical insensitivity of the first, third, and fourth natural frequencies to changes in the design parameter. The probabilistic moments $E\left(\omega_{2}\right)$, $\alpha \left(\omega_{2}\right)$, $E\left(\gamma_{2}\right)$, and $\alpha \left(\gamma_{2}\right)$ are presented illustratively in turn in Figure 12 and Figure 13.

Finally, the analysis of the influence of the random parameter *α_d_* on dynamic characteristics will be considered. The results are summarized in Table 11 and Table 12, respectively.

**Figure 14:**
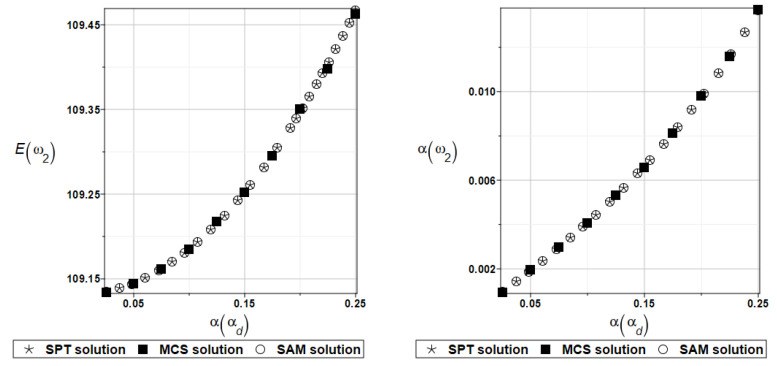
Probabilistic moments for the second natural frequency and the random distribution of the damper’s material parameter *α_d_*.

**Figure 15:**
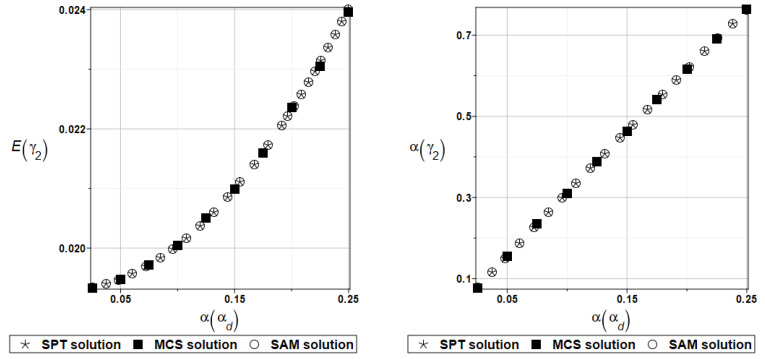
Probabilistic moments for the coefficient of damping and the random distribution of the damper’s material parameter *α_d_*.

To illustrate the random behavior of the solutions, the selected probabilistic moments $E\left(\omega_{2}\right)$, $\alpha \left(\omega_{2}\right)$, $E\left(\gamma_{2}\right)$, and $\alpha \left(\gamma_{2}\right)$ are presented in turn in Figure 14 and Figure 15.

**Figure 16:**
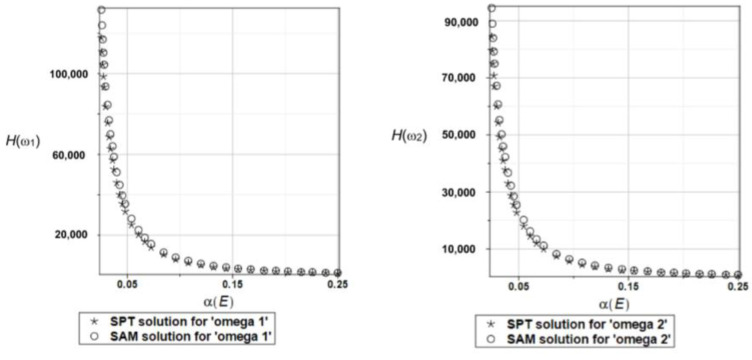
The probabilistic relative entropy for the random distribution of Young’s modulus.

**Figure 17:**
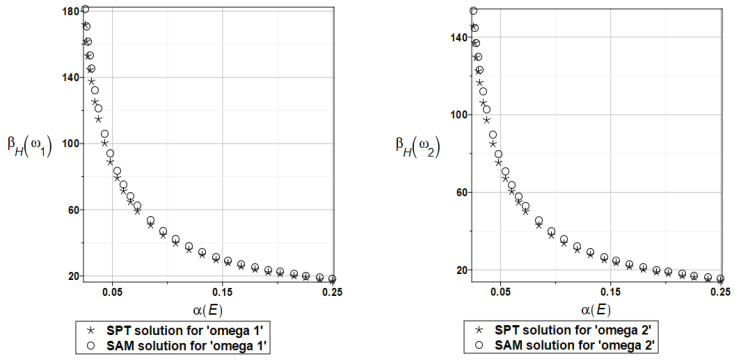
The probabilistic safety measure for the random distribution of Young’s modulus.

Probabilistic relative entropy *H* and the safety measure *β_H_* are estimated for the semi-analytical (SAM) and the stochastic perturbation technique (SPT) approaches for selected random parameters according to the relations Equations (27) and (28), and presented in Figure 16 and Figure 17, respectively.

Quite expectedly, both relative entropies and the following reliability indices decrease in an exponential way while increasing the input uncertainty, which is typical for the FORM-based indices in engineering applications. The plate is quite rationally designed, even with the viscoelastic supports, because even with extreme uncertainty in Young’s modulus reliability index is much larger than the widely used minimum values close to the interval [3.5, 5.2]. A little bit larger safety is noticed for the first eigenfrequency than for the second one.

### 4.2. Plate 2

**Figure 18:**
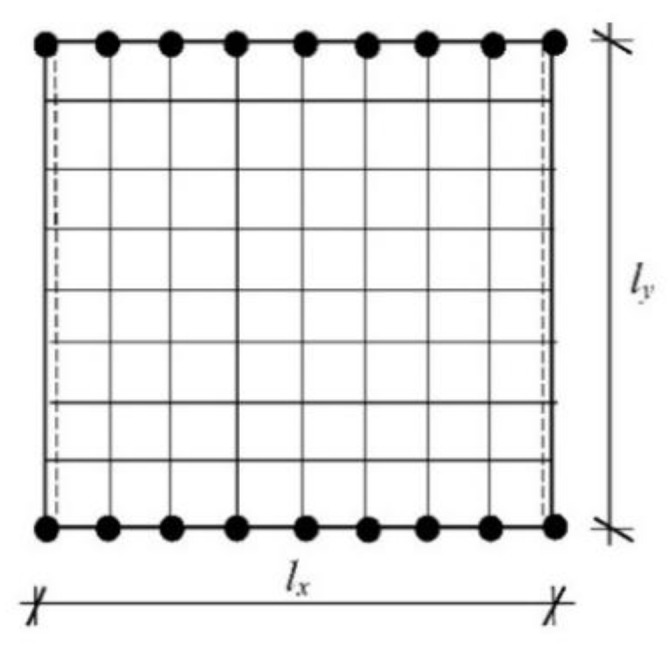
Finite element mesh of the square isotropic plate simply supported on two opposite edges, with the remaining edges resting on viscoelastic constraints.

The plate simply supported on two opposite edges with the other two resting on viscoelastic constraints is considered (Figure 18). The dimensions of the plate are identical to those adopted in the previous section. The influence of random physical and geometric parameters will be presented in a similar way as above, but it has been contrasted against the well-known analytical solution available in the literature. The first four eigenfrequencies determined with the mean value of Young’s modulus equal here in turn *ω* = {76.3133, 190.7834, 190.7834, 305.2534} [rad/s]. It is documented that an introduction of the nonlinear supports decreases almost twice the basic eigenfrequencies. This means that modeling errors consisting of an assumption that simple supports are assumed instead of real viscoelastic may lead to the structural danger of a resonance.

**Figure 19:**
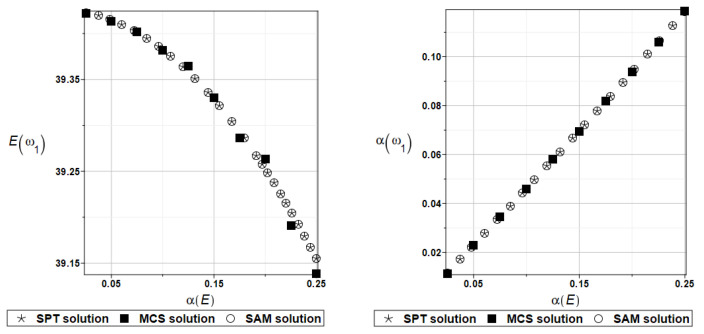
Probabilistic moments for the first natural frequency and the random distribution of Young’s modulus.

**Figure 20:**
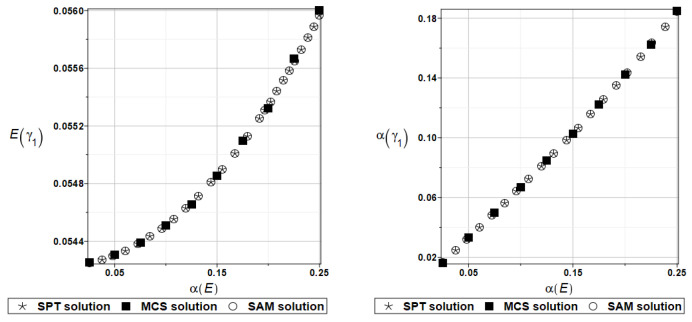
Probabilistic moments for the first coefficient of damping and the random distribution of Young’s modulus.

Probabilistic computations are carried out for the material properties of the plate and parameters describing viscoelastic supports whose values change randomly identically as for the plate presented in the preceding section. Identically, as in the previous examples, the set of deterministic solutions for the first four natural circular frequencies and coefficients of damping is given. The results are summarized in Table 13 and Table 14, respectively. Probabilistic characteristics, namely expected values and coefficients of variation, $E\left(\omega_{1}\right)$, $\alpha \left(\omega_{1}\right)$, $E\left(\gamma_{2}\right)$, and $\alpha \left(\gamma_{2}\right)$ are presented illustratively in turn in Figure 19 and Figure 20.

A set of deterministic solutions for the first four natural circular frequencies and coefficients of damping was determined. The results are summarized in Table 15 and Table 16, respectively.

**Figure 21:**
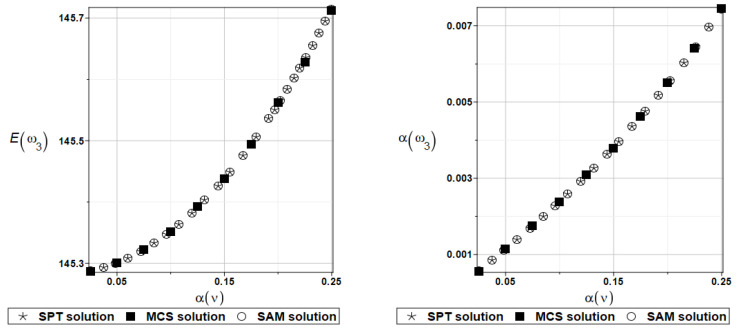
Probabilistic moments for the third natural frequency and the random distribution of Poisson’s ratio.

**Figure 22:**
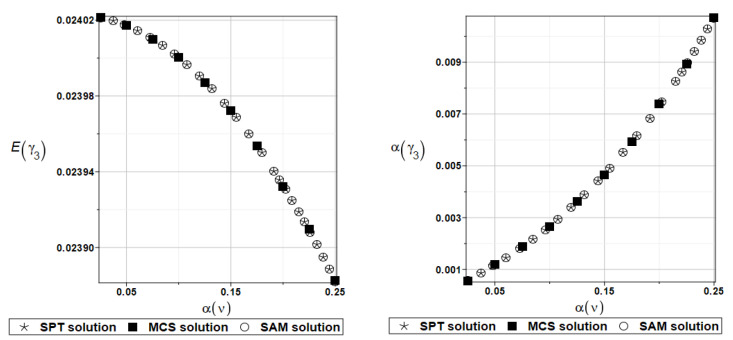
Probabilistic moments for the third coefficient of damping and the random distribution of Poisson’s ratio.

The probabilistic moments $E\left(\omega_{3}\right)$, $\alpha \left(\omega_{3}\right)$, $E\left(\gamma_{3}\right)$, and $\alpha \left(\gamma_{3}\right)$ are presented illustratively in turn in Figure 21 and Figure 22.

**Figure 23:**
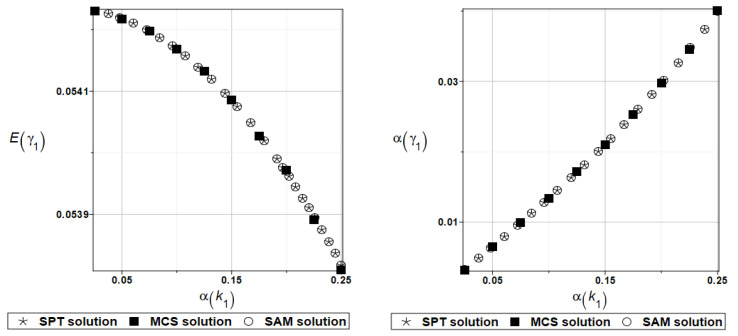
Probabilistic moments for the first coefficient of damping and the random distribution of the damper’s parameter *k*_1_.

Identically, as in the previous examples, the set of deterministic solutions for the first four natural circular frequencies and coefficients of damping is given. The results are summarized in Table 17 and Table 18, respectively. One can observe here the practical insensitivity of the solution to the design parameter *k*_0_. Furthermore, deterministic solutions for the first four natural circular frequencies and coefficients of damping are given. The results are summarized in Table 19 and Table 20, respectively. One can observe the practical insensitivity of the solution for circular frequencies to the design parameter *k*_1_. For illustration, the probabilistic moments $E\left(\gamma_{1}\right)$ and $\alpha \left(\gamma_{1}\right)$ have been presented in Figure 23. Deterministic solutions series for the first four natural circular frequencies and coefficients of damping are given. The results are summarized in Table 21 and Table 22, respectively.

**Figure 24:**
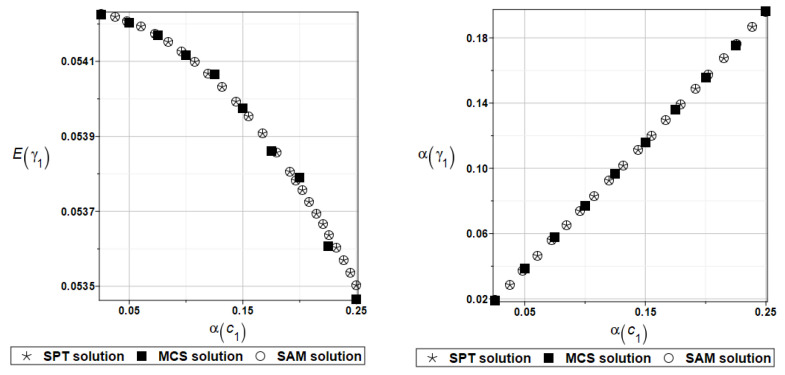
Probabilistic moments for the first coefficient of damping and the random distribution of the damper’s parameter *c*_1_.

One can observe here the total insensitivity of the solution for circular frequencies to the design parameter *c*_1_. For illustration, the probabilistic moments $E\left(\gamma_{1}\right)$ and $\alpha \left(\gamma_{1}\right)$ are presented in Figure 24. Determination of the response polynomial bases for the first four natural circular frequencies and coefficients of damping is given using the data collected in Table 23 and Table 24, respectively.

**Figure 25:**
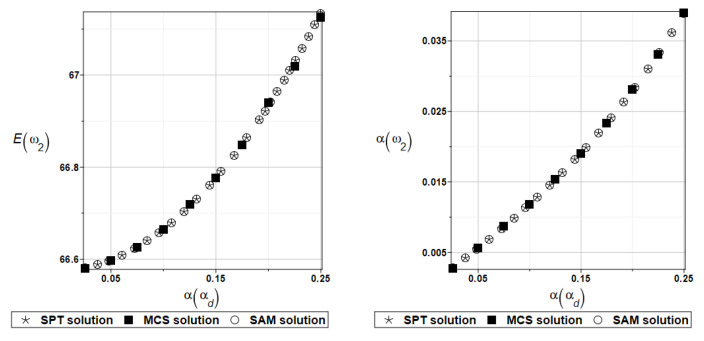
Probabilistic moments for the second natural frequency and the random distribution of the damper’s material parameter *α_d_*.

**Figure 26:**
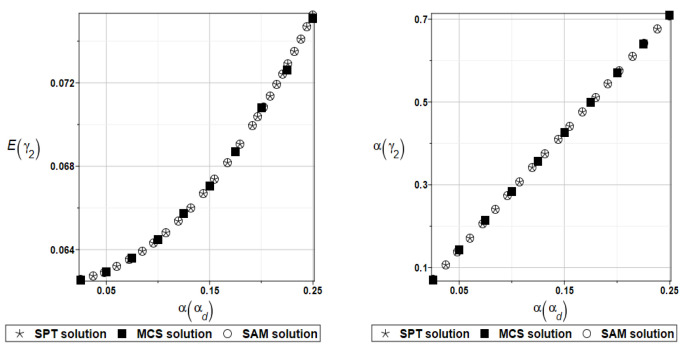
Probabilistic moments for the second coefficient of damping and the random distribution of the damper’s material parameter *α_d_*.

For an illustration of the structural random behavior, the probabilistic moments $E\left(\omega_{2}\right)$, $\alpha \left(\omega_{2}\right)$, $E\left(\gamma_{2}\right)$, and $\alpha \left(\gamma_{2}\right)$ are presented in Figure 25 and Figure 26.

**Figure 27:**
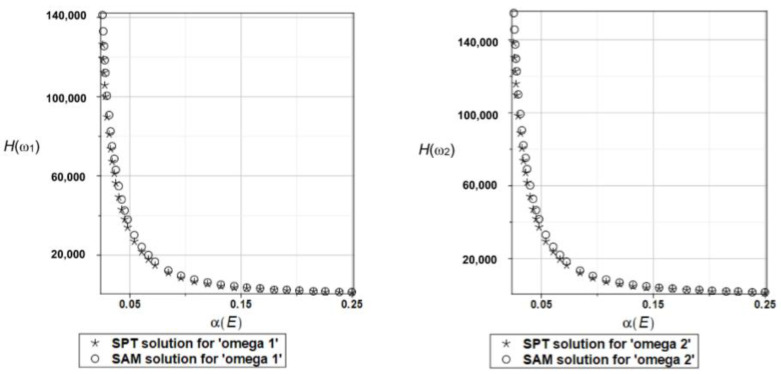
The probabilistic relative entropy for the random distribution of Young’s modulus.

**Figure 28:**
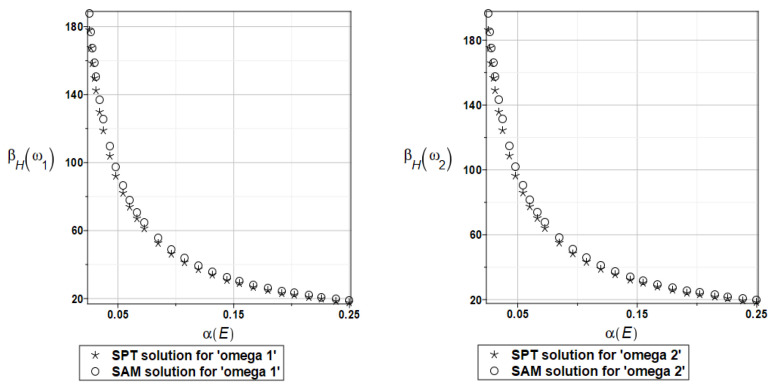
The probabilistic safety measure for the random distribution of Young’s modulus.

Probabilistic relative entropy *H* and the safety measure *β_H_* are estimated for the semi-analytical (SAM) and the stochastic perturbation technique (SPT) approaches for selected random parameters according to the relations Equations (27) and (28), and presented in Figure 27 and Figure 28, respectively.

Analogous observations hold true for Bhattacharyya entropy and the resulting reliability index as for the previous plate—both probabilistic methods return almost the same results—but now, both eigenfrequencies return almost the same target values and safety margin for the designed structure.

### 4.3. Plate 3

**Figure 29:**
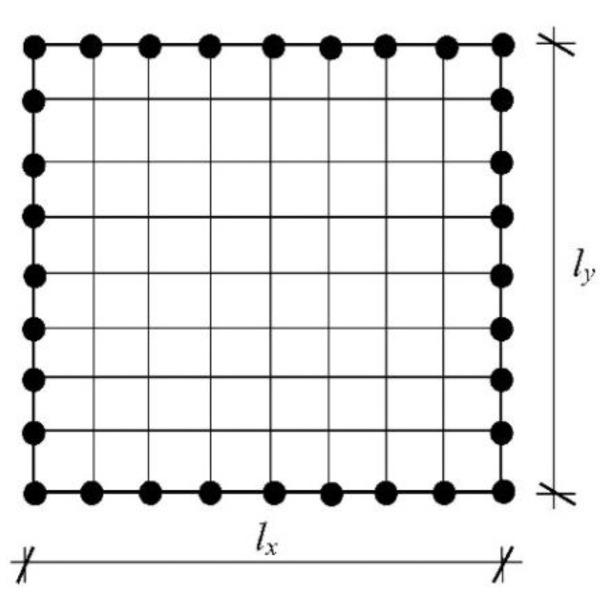
Finite element mesh of the square isotropic plate resting on viscoelastic constraints placed along all edges.

The square isotropic plate with all edges resting on viscoelastic constraints placed along all edges is considered (Figure 29). The influence of random physical and geometric parameters will be presented in a similar way as above.

Probabilistic computations are carried out for the material properties of the plate and parameters describing viscoelastic supports whose values change randomly identically as for the plate presented in Section 4.1. For this plate, the influence of material parameters is expressed by Young’s modulus *E*, Poisson’s ratio *v*, and the parameter *α_d_*. Identically, as in the examples presented previously, the set of deterministic solutions for the first four natural circular frequencies and coefficients of damping is given. The results are summarized in Table 25 and Table 26, respectively.

**Figure 30:**
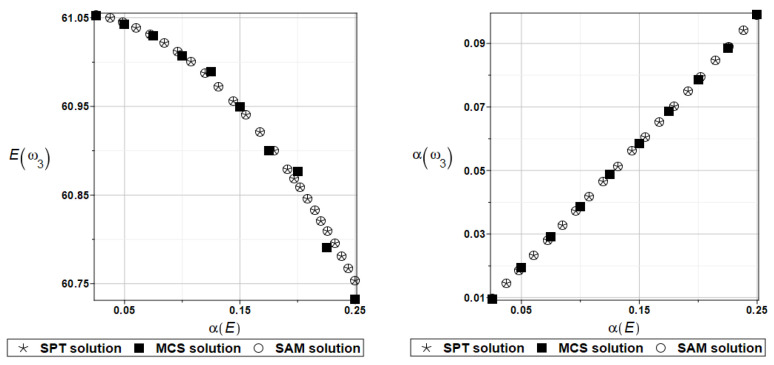
Probabilistic moments for the third natural frequency and the random distribution of Young’s modulus.

**Figure 31:**
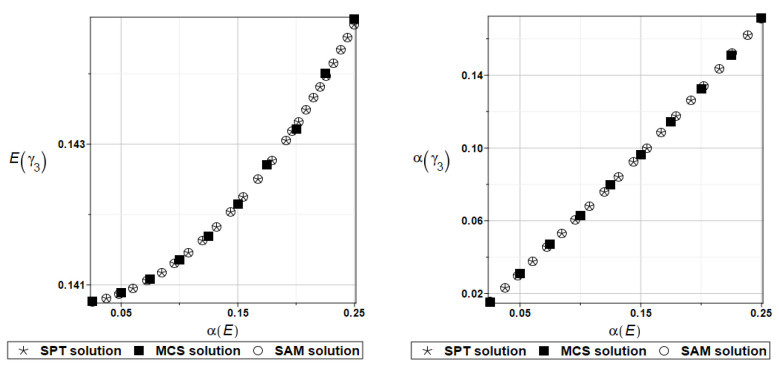
Probabilistic moments for the third coefficient of damping and the random distribution of Young’s modulus.

To illustrate the structural random behavior of a structure, the probabilistic moments $E\left(\omega_{3}\right)$, $\alpha \left(\omega_{3}\right)$, $E\left(\gamma_{3}\right)$, and $\alpha \left(\gamma_{3}\right)$ are presented in Figure 30 and Figure 31. Quite typically, for marginal resulting uncertainty in this problem, the expected values slightly decrease together with an increase in the input coefficient of variation, whereas the coefficient of variation of the eigenfrequencies increases in a linear manner.

**Figure 32:**
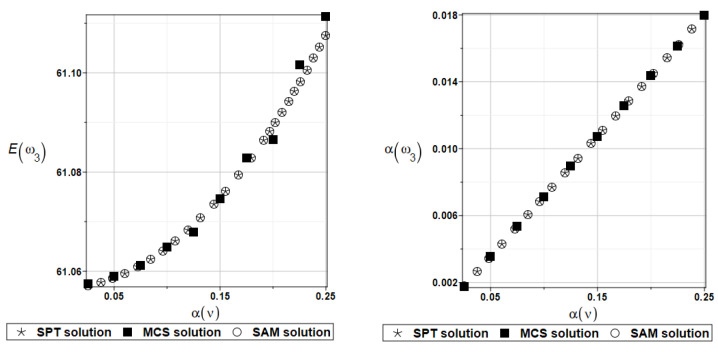
Probabilistic moments for the third natural frequency and the random distribution of Poisson’s ratio.

**Figure 33:**
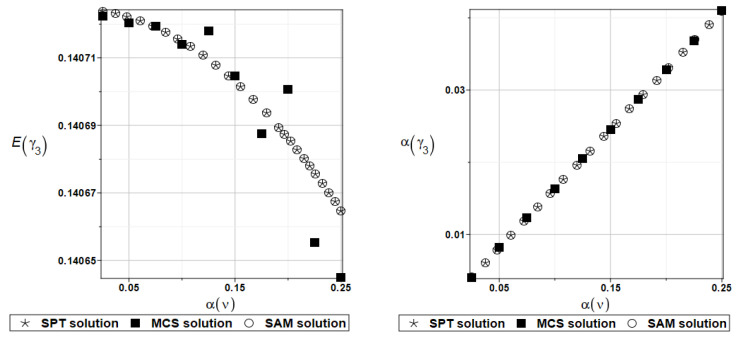
Probabilistic moments for the third coefficient of damping and the random distribution of Poisson’s ratio.

Probabilistic moments $E\left(\omega_{3}\right)$, $\alpha \left(\omega_{3}\right)$, $E\left(\gamma_{3}\right)$, and $\alpha \left(\gamma_{3}\right)$ following polynomial response functions approximated with the least squares method from the data contained in Table 27 and Table 28 are presented in Figure 32 and Figure 33 below.

**Figure 34:**
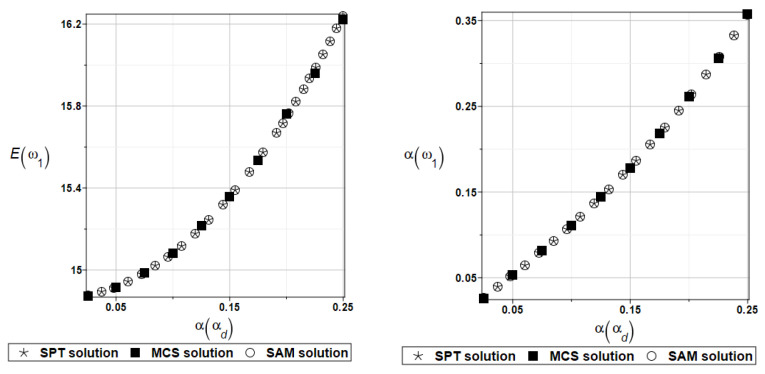
Probabilistic moments for the first natural frequency and the random distribution of the damper’s material parameter *α_d_*.

**Figure 35:**
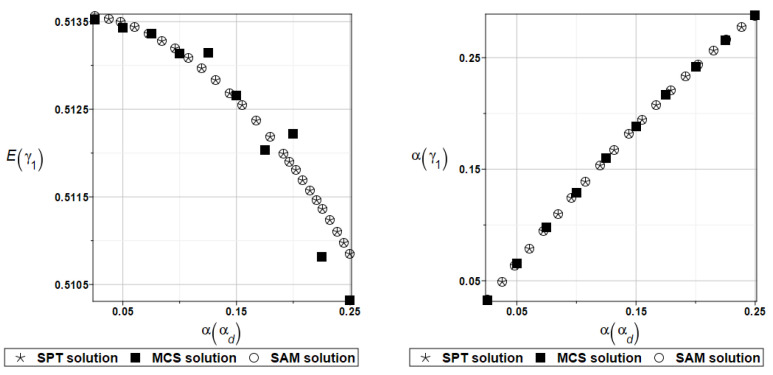
Probabilistic moments for the first coefficient of damping and the random distribution of the damper’s material parameter *α_d_*.

The same as previously, the set of deterministic solutions for the first four natural circular frequencies and coefficients of damping is given. The results are summarized in Table 29 and Table 30, respectively. Now, the sensitivity of the eigenfrequencies is definitely higher than before for the Poisson ratio. To illustrate the structural random behavior of a structure, the preselected probabilistic moments $E\left(\omega_{1}\right)$, $\alpha \left(\omega_{1}\right)$, $E\left(\gamma_{1}\right)$, and $\alpha \left(\gamma_{1}\right)$ are presented in Figure 34 and Figure 35. Contrary to Figure 32 and Figure 33, fluctuations of the expected values with respect to the input uncertainty are remarkable, whereas the resulting uncertainty level is a little bit larger than the input one. Therefore, the damper material has remarkable importance in safe design and in reliability analysis or prediction for thin plates with viscoelastic supports.

**Figure 36:**
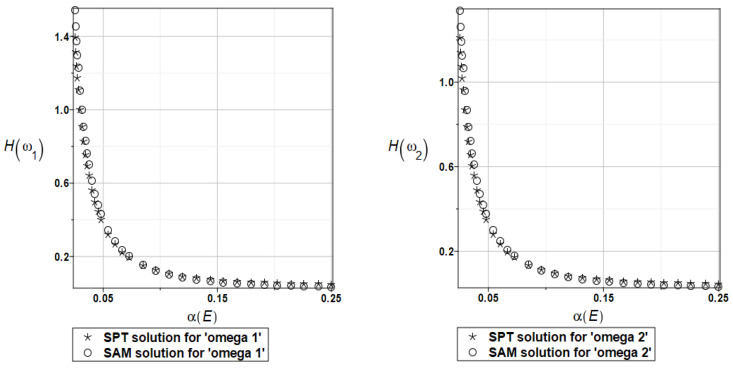
The probabilistic relative entropy for the random distribution of the damper’s material parameter *α_d_*.

**Figure 37:**
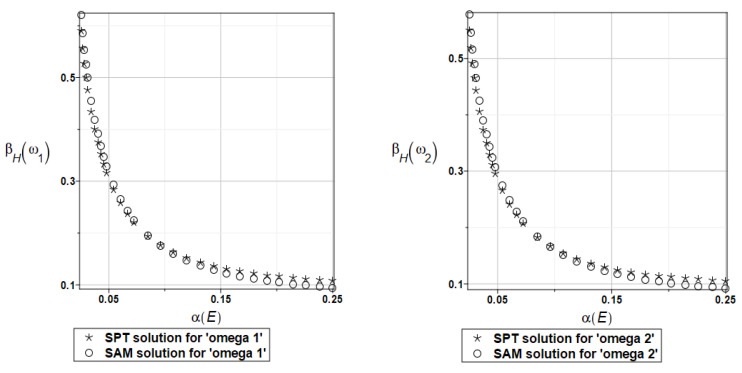
The probabilistic safety measure for the random distribution of the damper’s material parameter *α_d_*.

Similarly, as in the previous examples, the probabilistic relative entropy *H* and the safety measure *β_H_* are estimated for the semi-analytical (SAM) and the stochastic perturbation technique (SPT) approaches for selected random parameters according to the relations Equations (27) and (28), and presented in Figure 36 and Figure 37, respectively.

Now, the probabilistic entropy and the reliability index are defined on their basis, although they have quite typical distributions widely seen in the reliability theory. Nevertheless, their values are so small that even for minimum input uncertainty in the damper’s material parameter *α_d_*, these values are remarkably smaller than the values recommended in the engineering design as the admissible minimum. This once more confirms the paramount importance of this specific design parameter in the rational design of the plates under consideration.

## 5. Concluding Remarks

1. Fundamental eigenfrequencies of the Kirchhoff rectangular plates with various boundary conditions, including some viscoelastic supports, were studied in this paper. It was documented that viscoelastic supports dramatically decrease these eigenfrequencies with respect to plates having classical supports. The highest sensitivity of the solutions with respect to the viscoelastic support coefficients are documented; material parameters of the plate have remarkably smaller effect on the dynamic characteristics of the same plates. Therefore, uncertainty in the viscoelastic supports of the plates is decisive for their safety, reliability, and durability. The significance of viscoelasticity in supporting systems is so huge that all plates’ eigenfrequencies are completely insensitive to their elastic parameters, unlike in the classical elasticity. It can also be noticed that the use of non-integer order calculus allows for a good approximation of the description of the behavior of the given constraints, but to solve the nonlinear eigenproblem, it is necessary to use the so-called continuation method. Hence, here, the approach with state variables by definition cannot be used. It gains remarkable importance in structural problems with a relatively large number of degrees of freedom.
2. Theoretical and computational studies presented in this work clearly show that the common application of three probabilistic approaches, namely the semi-analytical (SAM), perturbation-based (SPT), and Monte-Carlo simulation technique (MCS) with continuation methods, allows for accurate determination of the probabilistic coefficients of free vibrations in the presence of input Gaussian uncertainty. In most cases, very good agreement in-between these three methods was noticed. Solving these problems consists primarily of determining the expected values and coefficients of variation for the circular natural frequencies along with the corresponding values of damping coefficients. Additionally, higher-order random moments are determined in selected problems, i.e., skewness and kurtosis, to verify if the desired output may have Gaussian PDF and ifverification is negative here. It must be mentioned that the third-degree response polynomials have been detected in all case studies as the most optimal approximation in-between structural output and input. This causes a maximum correlation coefficient and minimizes the mean square error of such a polynomial approximation.
3. From the engineering point of view, an insertion of further flexible nodes significantly reduces the value of the basic dynamic characteristics, i.e., the natural frequencies of vibrations, compared to the system in which such constraints do not exist. The value of the first circular frequency of the third plate is more than twice as small as the corresponding value for the previous plate—Table 25 and Table 13. As documented here, a change in the design parameter does not always result in a significant change in the solution, e.g., the results for the first four natural frequencies for the random distribution of the damper’s parameter *k*_0_. The reliability measures expressed by the probabilistic relative entropy *H* and coefficient *β_H_* show that their values are decreasing deeply as the value of the uncertainty coefficient increases. The simplest random approach is the semi-analytical one, and it allows us to derive random moments in analytical terms. This is relatively easy for future implementations in any academic and commercial finite element or other kind of computational programs.

## Figures and Tables

**Table 1:** The results for the first four natural frequencies and the random distribution of Young’s modulus.

*E* [GPa]	*ω*_1_ [rad/s]	*ω*_2_ [rad/s]	*ω*_3_ [rad/s]	*ω*_4_ [rad/s]
180	44.7706	102.4523	150.7459	214.4013
185	45.3388	103.8232	152.7976	217.3323
190	45.8995	105.1760	154.8220	220.2242
195	46.4532	106.5113	156.8200	223.0783
200	47.0002	107.8302	158.7926	225.8963
205	47.5406	109.1326	160.7410	228.6793
210	48.0748	110.4194	162.6658	231.4287
215	48.6030	111.6911	164.5679	234.1457
220	49.1252	112.9483	166.4482	236.8314
225	49.6418	114.1916	168.3073	239.4868
230	50.1528	115.4212	170.1460	242.1130

**Table 2:** The results for the first four coefficients of damping and the random distribution of Young’s modulus.

*E* [GPa]	*γ*_1_ [–]	*γ*_2_ [–]	*γ*_3_ [–]	*γ*_4_ [–]
180	0.052066	0.021361	0.008305	0.005605
185	0.051161	0.020908	0.008139	0.005482
190	0.050291	0.020476	0.007981	0.005364
195	0.049454	0.020064	0.007829	0.005252
200	0.048650	0.019667	0.007683	0.005145
205	0.047875	0.019290	0.007544	0.005043
210	0.047129	0.018929	0.007410	0.004945
215	0.046410	0.018583	0.007281	0.004851
220	0.045716	0.018251	0.007157	0.004761
225	0.045045	0.017931	0.007038	0.004674
230	0.044397	0.017624	0.006923	0.004591

**Table 3:** The results for the first four natural frequencies and the random distribution of Poisson’s ratio.

*V* [–]	*ω*_1_ [rad/s]	*ω*_2_ [rad/s]	*ω*_3_ [rad/s]	*ω*_4_ [rad/s]
0.25	47.2828	108.4706	159.0997	226.7159
0.26	47.3295	108.5801	159.4025	227.0638
0.27	47.3786	108.7008	159.7179	227.4336
0.28	47.4302	108.8332	160.0461	227.8258
0.29	47.4842	108.9769	160.3871	228.2409
0.30	47.5406	109.1326	160.7410	228.6793
0.31	47.5996	109.3004	161.1078	229.1417
0.32	47.6609	109.4808	161.4877	229.6286
0.33	47.7246	109.6741	161.8808	230.1408
0.34	47.7907	109.8805	162.2872	230.6790
0.35	47.8594	110.1006	162.7068	231.2439

**Table 4:** The results for the first four coefficients of damping and the random distribution of Poisson’s ratio.

*V* [–]	*γ*_1_ [–]	*γ*_2_ [–]	*γ*_3_ [–]	*γ*_4_ [–]
0.25	0.046118	0.019375	0.007118	0.004985
0.26	0.046461	0.019367	0.007198	0.004999
0.27	0.046808	0.019354	0.007281	0.005012
0.28	0.047160	0.019337	0.007366	0.005024
0.29	0.047516	0.019316	0.007454	0.005034
0.30	0.047875	0.019290	0.007544	0.005043
0.31	0.048240	0.019260	0.007637	0.005050
0.32	0.048610	0.019224	0.007732	0.005056
0.33	0.048984	0.019183	0.007831	0.005059
0.34	0.049363	0.019137	0.007932	0.005061
0.35	0.049748	0.019086	0.008036	0.005062

**Table 5:** The results for the first four natural frequencies and the random distribution of damper’s parameter *k*_0_.

*K*_0_ [N/m]	*ω*_1_ [rad/s]	*ω*_2_ [rad/s]	*ω*_3_ [rad/s]	*ω*_4_ [rad/s]
95	47.5200	109.1197	160.7347	228.6742
98	47.5241	109.1223	160.7360	228.6752
101	47.5283	109.1248	160.7372	228.6762
104	47.5324	109.1274	160.7385	228.6772
107	47.5365	109.1300	160.7397	228.6783
110	47.5406	109.1326	160.7410	228.6793
113	47.5448	109.1351	160.7422	228.6803
116	47.5489	109.1377	160.7435	228.6814
119	47.5531	109.1403	160.7447	228.6824
122	47.5572	109.1429	160.7459	228.6834
125	47.5612	109.1454	160.7472	228.6845

**Table 6:** The results for the first four coefficients of damping and the random distribution of the damper’s parameter *k*_0_.

*K*_0_ [N/m]	*γ*_1_ [–]	*γ*_2_ [–]	*γ*_3_ [–]	*γ*_4_ [–]
95	0.047944	0.019290	0.007546	0.005043
98	0.047931	0.019290	0.007546	0.005043
101	0.047917	0.019290	0.007545	0.005043
104	0.047903	0.019290	0.007545	0.005043
107	0.047889	0.019290	0.007544	0.005043
110	0.047875	0.019290	0.007544	0.005043
113	0.047862	0.019290	0.007543	0.005043
116	0.047848	0.019290	0.007543	0.005043
119	0.047834	0.019290	0.007542	0.005043
122	0.047821	0.019291	0.007542	0.005043
125	0.047807	0.019291	0.007541	0.005043

**Table 7:** The results for the first four natural frequencies and the random distribution of the damper’s parameter *k*_1_.

*K*_1_ [N/m]	*ω*_1_ [rad/s]	*ω*_2_ [rad/s]	*ω*_3_ [rad/s]	*ω*_4_ [rad/s]
17,500	47.5417	109.1356	160.7358	228.6728
18,000	47.5415	109.1351	160.7371	228.6744
18,500	47.5414	109.1346	160.7382	228.6759
19,000	47.5411	109.1340	160.7393	228.6772
19,500	47.5410	109.1333	160.7402	228.6783
20,000	47.5406	109.1326	160.7410	228.6793
20,500	47.5404	109.1318	160.7417	228.6802
21,000	47.5400	109.1310	160.7423	228.6809
21,500	47.5397	109.1301	160.7428	228.6815
22,000	47.5394	109.1292	160.7433	228.6821
22,500	47.5391	109.1283	160.7437	228.6826

**Table 8:** The results for the first four coefficients of damping and the random distribution of the damper’s parameter *k*_1_.

*K*_1_ [N/m]	*γ*_1_ [–]	*γ*_2_ [–]	*γ*_3_ [–]	*γ*_4_ [–]
17,500	0.046892	0.018644	0.007229	0.004784
18,000	0.047109	0.018786	0.007298	0.004840
18,500	0.047315	0.018921	0.007363	0.004894
19,000	0.047511	0.019050	0.007426	0.004946
19,500	0.047698	0.019173	0.007486	0.004995
20,000	0.047875	0.019290	0.007544	0.005043
20,500	0.048046	0.019403	0.007599	0.005088
21,000	0.048208	0.019510	0.007652	0.005132
21,500	0.048363	0.019614	0.007703	0.005175
22,000	0.048512	0.019713	0.007752	0.005215
22,500	0.048654	0.019808	0.007799	0.005254

**Table 9:** The results for the first four natural frequencies and random distribution of the damper’s parameter *c*_1_.

*C*_1_ [N·s/m]	*ω*_1_ [rad/s]	*ω*_2_ [rad/s]	*ω*_3_ [rad/s]	*ω*_4_ [rad/s]
205	47.2923	108.9110	160.5999	228.5450
210	47.3419	108.9552	160.6283	228.5721
215	47.3915	108.9995	160.6566	228.5990
220	47.4413	109.0439	160.6848	228.6258
225	47.4910	109.0882	160.7129	228.6526
230	47.5406	109.1326	160.7410	228.6793
235	47.5904	109.1769	160.7689	228.7059
240	47.6401	109.2209	160.7968	228.7324
245	47.6899	109.2653	160.8245	228.7588
250	47.7397	109.3096	160.8522	228.7851
255	47.7895	109.3540	160.8798	228.8114

**Table 10:** The results for the first four coefficients of damping and the random distribution of the damper’s parameter *c*_1_.

*C*_1_ [N·s/m]	*γ*_1_ [–]	*γ*_2_ [–]	*γ*_3_ [–]	*γ*_4_ [–]
205	0.043901	0.017609	0.006994	0.004676
210	0.044718	0.017953	0.007108	0.004753
215	0.045524	0.018293	0.007220	0.004827
220	0.046319	0.018629	0.007330	0.004901
225	0.047103	0.018962	0.007438	0.004972
230	0.047875	0.019290	0.007544	0.005043
235	0.048638	0.019615	0.007647	0.005112
240	0.049389	0.019939	0.007749	0.005179
245	0.050129	0.020256	0.007848	0.005246

**Table 11:** The results for the first four natural frequencies and the random distribution of the damper’s material parameter *α_d_*.

*α_d_* [–]	*ω*_1_ [rad/s]	*ω*_2_ [rad/s]	*ω*_3_ [rad/s]	*ω*_4_ [rad/s]
0.50	47.0136	108.5517	160.3107	228.2408
0.52	47.1075	108.6520	160.3828	228.3133
0.54	47.2065	108.7594	160.4612	228.3925
0.56	47.3112	108.8746	160.5464	228.4793
0.58	47.4223	108.9986	160.6393	228.5745
0.60	47.5406	109.1326	160.7410	228.6793
0.62	47.6672	109.2774	160.8525	228.7950
0.64	47.8032	109.4356	160.9752	228.9230

**Table 12:** The results for the first four coefficients of damping and the random distribution of the damper’s material parameter *α_d_*.

*α_d_* [–]	*γ*_1_ [–]	*γ*_2_ [–]	*γ*_3_ [–]	*γ*_4_ [–]
0.50	0.029935	0.011166	0.004429	0.002886
0.52	0.033009	0.012512	0.004959	0.003249
0.54	0.036325	0.013988	0.005534	0.003646
0.56	0.039898	0.015604	0.006156	0.004077
0.58	0.043743	0.017368	0.006826	0.004543
0.60	0.047875	0.019290	0.007544	0.005043
0.62	0.052313	0.021384	0.008309	0.005576
0.64	0.057071	0.023653	0.009119	0.006141
0.66	0.062168	0.026109	0.009969	0.006733
0.68	0.067617	0.028761	0.010855	0.007347
0.70	0.073437	0.031612	0.011767	0.007974

**Table 13:** The results for the first four natural frequencies and the random distribution of Young’s modulus.

*E* [GPa]	*ω*_1_ [rad/s]	*ω*_2_ [rad/s]	*ω*_3_ [rad/s]	*ω*_4_ [rad/s]
180	37.1491	62.7840	136.4726	142.6124
185	37.6159	63.5616	138.2809	144.5536
190	38.0767	64.3293	140.0650	146.4688
195	38.5318	65.0877	141.8260	148.3591
200	38.9814	65.8369	143.5655	150.2255
205	39.4257	66.5773	145.2833	152.0689
210	39.8648	67.3093	146.9808	153.8900
215	40.2990	68.0330	148.6586	155.6896
220	40.7283	68.7489	150.3176	157.4686
225	41.1531	69.4570	151.9577	159.2275
230	41.5733	70.1577	153.5803	160.9671

**Table 14:** The results for the first four coefficients of damping and the random distribution of Young’s modulus.

*E* [GPa]	*γ*_1_ [–]	*γ*_2_ [–]	*γ*_3_ [–]	*γ*_4_ [–]
180	0.059028	0.068283	0.026588	0.008418
185	0.057991	0.067014	0.026025	0.008249
190	0.056991	0.065799	0.025490	0.008087
195	0.056039	0.064633	0.024980	0.007932
200	0.055120	0.063515	0.024490	0.007783
205	0.054235	0.062440	0.024023	0.007641
210	0.053383	0.061407	0.023575	0.007504
215	0.052562	0.060412	0.023144	0.007373
220	0.051770	0.059455	0.022730	0.007247
225	0.051005	0.058531	0.022334	0.007125
230	0.050267	0.057640	0.021951	0.007008

**Table 15:** The results for the first four natural frequencies and the random distribution of Poisson’s ratio.

*V* [–]	*ω*_1_ [rad/s]	*ω*_2_ [rad/s]	*ω*_3_ [rad/s]	*ω*_4_ [rad/s]
0.25	39.0949	66.9109	144.9199	150.4124
0.26	39.1571	66.8397	144.9639	150.7189
0.27	39.2213	66.7707	145.0217	151.0376
0.28	39.2874	66.7040	145.0944	151.3688
0.29	39.3555	66.6396	145.1812	151.7125
0.30	39.4257	66.5773	145.2833	152.0689
0.31	39.4978	66.5172	145.4009	152.4380
0.32	39.5719	66.4593	145.5336	152.8202
0.33	39.6480	66.4034	145.6829	153.2154
0.34	39.7262	66.3496	145.8481	153.6238
0.35	39.8064	66.2978	146.0307	154.0456

**Table 16:** The results for the first four coefficients of damping and the random distribution of Poisson’s ratio.

*V* [–]	*γ*_1_ [–]	*γ*_2_ [–]	*γ*_3_ [–]	*γ*_4_ [–]
0.25	0.052825	0.060718	0.024053	0.007281
0.26	0.053101	0.061062	0.024056	0.007349
0.27	0.053380	0.061407	0.024056	0.007419
0.28	0.053662	0.061751	0.024048	0.007491
0.29	0.053947	0.062096	0.024039	0.007565
0.30	0.054235	0.062440	0.024023	0.007641
0.31	0.054526	0.062785	0.024001	0.007719
0.32	0.054821	0.063130	0.023977	0.007799
0.33	0.055119	0.063475	0.023945	0.007881
0.34	0.055422	0.063820	0.023910	0.007965
0.35	0.055727	0.064166	0.023867	0.008051

**Table 17:** The results for the first four natural frequencies and the random distribution of the damper’s parameter *k*_0_.

*K*_0_ [N/m]	*ω*_1_ [rad/s]	*ω*_2_ [rad/s]	*ω*_3_ [rad/s]	*ω*_4_ [rad/s]
95	39.4043	66.5454	145.2648	152.0628
98	39.4086	66.5518	145.2685	152.0640
101	39.4129	66.5582	145.2723	152.0652
104	39.4171	66.5646	145.2760	152.0664
107	39.4214	66.5710	145.2798	152.0676

**Table 18:** The results for the first four coefficients of damping and the random distribution of the damper’s parameter *k*_0_.

*K*_0_ [N/m]	*γ*_1_ [–]	*γ*_2_ [–]	*γ*_3_ [–]	*γ*_4_ [–]
95	0.054315	0.062498	0.024024	0.007643
98	0.054299	0.062487	0.024024	0.007643
101	0.054283	0.062475	0.024023	0.007642
104	0.054267	0.062463	0.024023	0.007642
107	0.054251	0.062452	0.024022	0.007641

**Table 19:** The results for the first four natural frequencies and the random distribution of the damper’s parameter *k*_1_.

*K*_1_ [N/m]	*ω*_1_ [rad/s]	*ω*_2_ [rad/s]	*ω*_3_ [rad/s]	*ω*_4_ [rad/s]
17,500	39.4280	66.5811	145.2801	152.0650
18,000	39.4276	66.5805	145.2812	152.0660
18,500	39.4271	66.5798	145.2824	152.0669
19,000	39.4266	66.5791	145.2830	152.0676
19,500	39.4262	66.5782	145.2834	152.0683

**Table 20:** The results for the first four coefficients of damping and the random distribution of the damper’s parameter *k*_1_.

*K*_1_ [N/m]	*γ*_1_ [–]	*γ*_2_ [–]	*γ*_3_ [–]	*γ*_4_ [–]
17,500	0.053241	0.060875	0.023070	0.007332
18,000	0.053460	0.061220	0.023279	0.007399
18,500	0.053669	0.061547	0.023476	0.007464
19,000	0.053866	0.061859	0.023666	0.007525
19,500	0.054055	0.062157	0.023848	0.007584

**Table 21:** The results for the first four natural frequencies and the random distribution of the damper’s parameter *c*_1_.

*C*_1_ [N·s/m]	*ω*_1_ [rad/s]	*ω*_2_ [rad/s]	*ω*_3_ [rad/s]	*ω*_4_ [rad/s]
205	39.1985	66.1265	144.8963	151.9355
210	39.2438	66.2165	144.9740	151.9623
215	39.2892	66.3066	145.0515	151.9891
220	39.3346	66.3967	145.1286	152.0157
225	39.3801	66.4870	145.2062	152.0423

**Table 22:** The results for the first four coefficients of damping and the random distribution of the damper’s parameter *c*_1_.

*C*_1_ [N·s/m]	*γ*_1_ [–]	*γ*_2_ [–]	*γ*_3_ [–]	*γ*_4_ [–]
205	0.049568	0.057213	0.022088	0.007068
210	0.050524	0.058287	0.022486	0.007187
215	0.051468	0.059346	0.022879	0.007304
220	0.052402	0.060392	0.023267	0.007418
225	0.053324	0.061423	0.023647	0.007531

**Table 23:** The results for the first four natural frequencies and the random distribution of the damper’s material parameter *α_d_*.

*α_d_* [–]	*ω*_1_ [rad/s]	*ω*_2_ [rad/s]	*ω*_3_ [rad/s]	*ω*_4_ [rad/s]
0.50	38.9825	65.5297	144.1695	151.6698
0.52	39.0628	65.7132	144.3574	151.7370
0.54	39.1469	65.9082	144.5607	151.8098
0.56	39.2351	66.1161	144.7811	151.8888
0.58	39.3278	66.3385	145.0212	151.9749
0.60	39.4257	66.5773	145.2833	152.0689
0.62	39.5293	66.8349	145.5714	152.1719
0.64	39.6393	67.1142	145.8882	152.2851
0.66	39.7569	67.4187	146.2393	152.4098
0.68	39.8831	67.7527	146.6298	152.5474
0.70	40.0195	68.1218	147.0659	152.6997

**Table 24:** The results for the first four coefficients of damping and the random distribution of the damper’s material parameter *α_d_*.

*α_d_* [–]	*γ*_1_ [–]	*γ*_2_ [–]	*γ*_3_ [–]	*γ*_4_ [–]
0.50	0.034187	0.037911	0.013852	0.004476
0.52	0.037626	0.042057	0.015548	0.005011
0.54	0.041332	0.046559	0.017403	0.005594
0.56	0.045323	0.051441	0.019429	0.006226
0.58	0.049617	0.056726	0.021632	0.006908
0.60	0.054235	0.062440	0.024023	0.007641
0.62	0.059197	0.068610	0.026604	0.008425
0.64	0.064525	0.075263	0.029385	0.009258
0.66	0.070243	0.082423	0.032360	0.010137
0.68	0.076376	0.090117	0.035527	0.011059
0.70	0.082951	0.098368	0.038877	0.012016

**Table 25:** The results for the first four natural frequencies and the random distribution of Young’s modulus.

*E* [GPa]	*ω*_1_ [rad/s]	*ω*_2_ [rad/s]	*ω*_3_ [rad/s]	*ω*_4_ [rad/s]
180	14.8318	24.4221	58.0999	78.2479
185	14.8393	24.4260	58.7038	79.1780
190	14.8464	24.4292	59.3013	80.0965
195	14.8532	24.4319	59.8924	81.0043
200	14.8596	24.4350	60.4776	81.9014

**Table 26:** The results for the first four coefficients of damping and the random distribution of Young’s modulus.

*E* [Gpa]	*γ*_1_ [–]	*γ*_2_ [–]	*γ*_3_ [–]	*γ*_4_ [–]
180	0.509837	0.533552	0.152404	0.090064
185	0.510657	0.533790	0.149889	0.088395
190	0.511434	0.534026	0.147469	0.086796
195	0.512173	0.534264	0.145139	0.085262
200	0.512876	0.534475	0.142893	0.083790

**Table 27:** The results for the first four natural frequencies and the random distribution of Poisson’s ratio.

*v* [–]	*ω*_1_ [rad/s]	*ω*_2_ [rad/s]	*ω*_3_ [rad/s]	*ω*_4_ [rad/s]
0.25	14.8509	24.4341	61.8065	83.6013
0.26	14.8539	24.4350	61.6526	83.4333
0.27	14.8568	24.4358	61.5008	83.2680
0.28	14.8597	24.4358	61.3508	83.1054
0.29	14.8627	24.4369	61.2029	82.9455

**Table 28:** The results for the first four coefficients of damping and the random distribution of Poisson’s ratio.

*v* [–]	*γ*_1_ [–]	*γ*_2_ [–]	*γ*_3_ [–]	*γ*_4_ [–]
0.25	0.511905	0.534396	0.136860	0.080368
0.26	0.512231	0.534441	0.137637	0.080772
0.27	0.512558	0.534496	0.138412	0.081174
0.28	0.512886	0.534525	0.139185	0.081575

**Table 29:** The results for the first four natural frequencies and the random distribution of the damper’s material parameter *α_d_*.

*α_d_* [–]	*ω*_1_ [rad/s]	*ω*_2_ [rad/s]	*ω*_3_ [rad/s]	*ω*_4_ [rad/s]
0.50	12.7353	20.1901	58.7623	80.8862
0.52	13.0975	20.9059	59.1553	81.2122
0.54	13.4877	21.6804	59.5769	81.5621
0.56	13.9095	22.5215	60.0309	81.9388
0.58	14.3672	23.4371	60.5222	82.3461
0.60	14.8656	24.4378	61.0567	82.7882

**Table 30:** The results for the first four coefficients of damping and the random distribution of the damper’s material parameter *α_d_*.

*α_d_* [–]	*γ*_1_ [–]	*γ*_2_ [–]	*γ*_3_ [–]	*γ*_4_ [–]
0.50	0.401650	0.420408	0.087266	0.049563
0.52	0.423633	0.443153	0.096394	0.055101
0.54	0.445879	0.466047	0.106259	0.061121
0.56	0.468353	0.488975	0.116905	0.067653
0.58	0.490930	0.511909	0.128378	0.074727

## Data Availability

Data are contained within the article.
